# The Impact of Ethical Leadership on Employees’ Green Innovation Behavior: A Mediating-Moderating Model

**DOI:** 10.3389/fpsyg.2022.951861

**Published:** 2022-06-29

**Authors:** Liqin Yang, Haibing Liu

**Affiliations:** ^1^School of Economics and Management, Lanzhou Jiaotong University, Lanzhou, China; ^2^Evergrade School of Management, Wuhan University of Science and Technology, Wuhan, China

**Keywords:** green innovation behavior, ethical leadership, strategic flexibility, green organizational identity, SEM

## Abstract

To enhance environmental protection and sustainable development, green innovation (GI) is an inevitable choice for enterprises. This study incorporates social identity theory and social learning theory to explore the impact of ethical leadership on employee GI behavior. In addition, this study also examines the mediating effects of green organizational identity (GOI) and the moderating role of strategic flexibility (SF). Using the structural equation modeling, an empirical survey was conducted among 300 Chinese manufacturing companies. The study found that ethical leadership (EL) positively affects employees’ GI behavior (EGIB). It also positively impacts the GOI, which led to EGIB. In addition, the study also confirmed that GOI played a mediating role in the relationship between EL and EGIB. The results further indicated that SF positively enhanced the effect of GOI on EGIB. The findings have important contributions to theory and practice in the current research context.

## Introduction

There has been a huge rise in public awareness regarding the continued eminence of water fouling, haze, and global warming. The worry about environmental protection is also rising (Ma et al., 2021). With the rapid change in a typical condition of China’s financial system, the change drive and green development have developed the strongest ideas of national growth (Lee and Lee, 2022), which lead to the green change (Jänicke, 2012). The microelement of a major environmental ecosystem, the effectiveness, depends on society’s green resilience for firms. Considering green creation as the main essential planned instrument playing a role in the sustainable growth of firms (Xu et al., 2020), it cannot only bear the required challenges of environmental safety. To fulfill all the social duties and the functions that firms may fulfill and acknowledge the innovation regarding brand designing (Peters and Buijs, 2022) managers are playing a role in enhancing the competitive advantage of goods and helping firms improve presentation. Thus, acquiring the conventional strategies of creative ideas and concepts, adopting a general trend of the modern concept of green innovation (GI) (Begum et al., 2022), has become an essential tool for the development by the enterprisers with a win-win effect without considering the region or the industry practices of GI.

Individuals in enterprise firms have unique ideas and suggestions, which help solve all major issues (Wong and Aspinwall, 2004); along with this, they deal very efficiently with all company matters. Thus, the importance of GI is highly essential and eventually be implemented for employees to focus on their work (Jong, 2007; Epezagne Assamala et al., 2022). The positive GI behavior of an individual is quite important in the organization for better success. Recently, scholars and analysts have explored the impact of GI behavior in the various fields of life and society. The researchers concentrate on the influence of GI on the firm workability, presentation, and financial growth, specifically on the basic side of corresponding resources, which is considered a tactical tool for enhancing competitive advantages (Fu et al., 2017). The literary community has ever considered GI a revolutionary step. In recent years, researchers have concentrated on the advantages of sustainable innovation and succeeded a lot in achieving research results (Schot and Geels, 2008; Mangenda Tshiaba et al., 2021). Previously, it was studied that R&D financing, industrial accumulation, administrative learning, and ecological regulations affect sustainable innovation (Ketata et al., 2015).

Moreover, certain analysts also pay attention to the environmental awareness of firms’ resources, executives, excessive, and variables as the moderate factors to evaluate the affecting elements of GI (Li G. et al., 2020). Therefore, analyzing the factors that impact GI behavior may bring change individually, organizationally, and socially. Ethical leadership (EL) plays a significant role in enterprises. It is the most influential element to which the employees are exposed (Sherehiy et al., 2007; Qader et al., 2022) and also influences the employee’s GI behavior. However, the studies about the impact on organizational behavior are few. This research focuses on the theory of planned behavior, comprising various variables of individuals of GI, behavior norms, endogenous attitude, and revolution readiness (Ataei et al., 2021). Some analysts explain the effect of the mechanism of entente bags on green change behavior based on several dossiers. Ethical heads practice ethics and show employee about the behavior interaction (Wimbush and Shepard, 1994) and appropriate ethics practically and in interpersonal relations to encourage the individuals to show moral behavior in two imparting for reasons and other control strategies (Akhmetshin et al., 2020). A moral leader focuses on employees to lead them to sustainability. Ethical management has been a topic of serious issue in recent times (Nuseir and Ghandour, 2019). Particularly in the aspect of the Chines setting, the moral properties are in front. As per the “social learning model,” individuals’ GI response and behavior may be highly affected by management behavior (Liu and Zhao, 2019). Thus, regarding leadership behavior, it is important to find the nexus between moral management and workers’ green change behavior which is the major problem (Saleem et al., 2020) that needs to be discussed in the following research.

The second problem is the middle process between EL and workers’ green change behavior (Li et al., 2021). Thus, evaluating the conceptual framework of the connection between ethical management and the employees’ GI behavior (EGIB) has used fluid of green organizational recognition to facilitate explaining the association among them in a clear way (Malik et al., 2021) and organizational performance in other aspects (Shahzad et al., 2022). Management behavior is a major factor that causes a great change in the psychological state of employees. Leadership behavior, along with the mental condition of staff members, will bring change in their attitude and behavior (Bommer et al., 2005). So, it is essential to combine management behavior with the employee’s mental state. Organizational recognition is a factor that helps reflect the extent to which individuals of an organization recognize their level of internalization of the organization’s values and goals (Dickson et al., 2001). Such variables help not only to evaluate the employee’s behavior but also explore the employee’s psychological relationship with the organization and their loyalty. Though many studies have been performed regarding directorial identity, there is still more to be studied about green directorial identity, which tends to combine organizational recognition with innate ecological elements and implement environmental management (Ik and Azeez, 2020). Hence, in this study, we explained the mediating part of green organizational identity (GOI) between moral leadership and individuals’ GIB.

In this study, the third major issue is that there are certain limits and border states between moral leadership and employee GIB, so we should focus on the specific properties of both (Singh et al., 2020). Thus, we should focus on the boundary characteristics that may affect the association between ethical management and employees’ GIB called planned efficiency. It introduces individuals’ recognized strategic elasticity to calculate workers’ attitudes toward management behavior in this regard. This research determines the responses like “under what circumstances EL can play a better role to motivate employees engaging in GI.” Hence, this study considers strategic efficiency as the boundary state to influence the connection between ethical management and employees’ GIB. Compared to past research, this study largely follows the contrasts. The first is that the resultant factor discussed in this study is not the green revolution presentation, yet the GIB of workers, reexamining the green revolution from a single perspective. Next, strategic efficiency is mainly explained in the study model, and extra focus is paid to an individual’s perceived planned efficiency. With the help of the above-explained discussion and evaluation, the research aims to evaluate the value of moral management that influences individuals’ GIB and whether green directorial identity plays a conciliating part in evaluating strategic efficiency as an arbitrating effect.

## Literature Review and Hypotheses Development

Leadership as behavior; leaders are models to set an example of personal and interpersonal communication with the lower and working staff. Moral behavior is through face-to-face conversation, motivation, and power of handling issues (Burkhalter et al., 2002). The scholars mentioned that moral guidance comprises two major traits. First is the moral discrete comprising honesty and integrity, carries them in daily life, and abides by the basic ethical norms. The second is the ethical manager, leading with the quality of benefiting the lower staff, establishing moral values to the working members through practice (Hammann et al., 2009), communication through effective mode exemplifying the subordinate to show behavioral ethics and morality (Lin et al., 2016), and taking moral decisions influencing the firm’s behavior and ethical norms. Furthermore, technological advancements have brought the digital and physical worlds together, as well as the potential to make cognitive judgments without the intervention of humans (Hu et al., 2022). Ethical issues related to leadership may arise due to such topical involvement of technology in the manufacturing sector.

The domestic experts have evaluated and studied the very strong influential elements and circumstances of moral guidelines from the communal culture hypothesis perspective, individuality characteristics (Smith and Hume, 2005), and the moral uniqueness theory (Abosag et al., 2020). Karsh oven supposed that the firmness of character traits plays well in predicting moral management. The big five personality traits mentioned by the scholars have mentioned a sense of responsibility, agreeableness, and a stable emotional condition positively affecting the EL (Liu and Zhao, 2019). The research mentioned very clearly the superior the morality or the social norms and values of superiors are, the socially and morally the positive impact on moral values (Schwepker, 2017). Regarding moral traits, the study found that ethically strong leadership may enhance the employees’ level of satisfaction, their creative skills, and advisory behavior (Yidong and Xinxin, 2013), promotion of commercial social responsibility and another optimistic attitude, increase employees’ innovative behavior, and quit the negative behavior (Lee et al., 2021).

Based on social culture theory, the study expresses that moral guidance impacts staff effort very positively, known as ecological behavior, to show a very deep association between moral guidance and workforce ecological novelty identity. Though ecological innovation impacts very positively, it has not had such a positive impact on all the employees collectively as there are situational characteristics between them. Facing various uncertain circumstances or factors, employees may face highly uncertain conditions or situations. Ecological modernism is doubtful and very perilous to adopt deliberately. Planned elasticity presents the energetic quality of the venture under such doubtful situations. Consequently, the following research hands out tactical suppleness into the structure as a limitation or the edge to evaluate and analyze the response and behavior variations and disparity of staff under various awareness stages and constructs and builds up a hypothetical form comprising variables, namely, workforce ecological revolutionary behavior, calculated flexibility, and moral guidance.

### Ethical Leadership and Employee’s Green Innovation Behavior

Green revolution helps reduce ecological pollutant loss by making the green item and products and defining procedures and setups (Armanda et al., 2019), and developing the process and systems regarding energy protection and contamination anticipation and organization, and waste recycling. Many scholars and researchers have regarded GI as a strong element in increasing spirited benefits and enhancing business image and values (Gallardo-Vázquez et al., 2019). Experts have analyzed the benefits of using the innovation effects or advantages in transformational leadership and environmental organization (García-Morales et al., 2008). The word environment is multidimensional. Organizations as support between firms and staff have a significant influence on working staff behavior and response (Yoon and Suh, 2003); when the guidance has the power of significance, the subordinate has been considered a strength. Regarding the Chinese moral context, ethical guidance is a valuable form of leading that will impact human resources green chains behavior (Bai et al., 2019) just by the ethical management and the employee’s performance (Tan, 2009). First, just headship is not considered a right entity, but a moral expert; this spirit of responsibility, moral values, and obligations is more likely relevant to staff behavior.

Strongly motivated managers will influence positively by developing very positive employee ethics. Moral guideline asks for attention to oral administration. A leader must be skilled in honest management, and event working will surely pay concentration to strengthen and focus on community liability to R&D and promote green fabrication and guide to give confidence staff to investigate and bring out the pioneering outcome with green advantages (Hughes and Terrell, 2011). Hence, EL cannot be estranged from the accomplishment of moral leadership quality and morality. Second, social culture opinion also highlights workforce understanding and practice by overviewing their supervisor’s behavior pattern. Influential with qualities of ethical morality is an example for the employees and source of information (Lei et al., 2019).

Realizing ethical values such as caring about others enhances their attention. They pay more concentration to more reliable progress and possess the ethics and qualities of such innovation in production. This empowers the employees to be more innovative and creative in leading others. This will help develop a social relationship with their leaders (Hoch, 2013). Moral behavior, innovative inspiration, and other behaviors as their behavior models carry out GI behavior very actively. Furthermore, the following research expresses that moral guidance will surely contact staff and the firms positively to encourage the workers to behave positively. In contrast, green revolution behavior is a very optimistic and vigorous behavior of working staff not just following the rules and system within the organization but also in social life (DuBrin, 2013).

It has the basic ground with public organizational behaviors, creativity, and other positive behavior. Moral guidance will surely be more accountable for moral decisions, contribute to answerable social actions and ecological fortification, and from side-to-side-related organization incentive, and enhances the working staff support for green chains activities so that the staff can contribute to them. In contrast to the other management behaviors, moral guidance is green and reliable implementer to recognize the different requirements of the workforce for GI and promote innovation using resources distribution personalized services. According to the study, the following hypothesis has been proposed:

**Hypothesis 1:** EL has a positive and significant impact on EGIB.

### Ethical Leadership, Green Organizational Identity, and Employees’ GI Behavior

The organizational identity impacts the level of internationalization of workers’ perception of their norms and objectives. The study indicates that the “organizational identity” can reflect the employee’s attitude and behavior (Cole and Bruch, 2006) to understand the nature of green safety behavior. The individuals of the firm can develop an “organizational identity” framework relating to ecological revolution and environmental control (Chang and Hung, 2021). That is an ecological directorial identity. Moral managers care regarding the benefits of individuals, facilitate them in solving issues and problems, are keener to establish continual growth of firms, and have a powerful concept of morals and CSR. Firms with an ethical environment help regulate the system and develop a strong image. After perceiving the employees that their leaders have the ethical qualities, they will surely feel the pride to perform their duties (Kim and Brymer, 2011) and work in such a satisfying place, which helps in reducing the social-psychological distance between the leaders and the employees, who win their loyalty and sincerity for the organization (Hernández-Ortega, 2018).

When there is no social and environmental gap between the employees and the leaders in the organization (Robertson and Barling, 2013), this will affect the employee’s performance and environmental protection and realization of the environmental goals for the organization (Chen et al., 2015). Such a positive environment helps the leaders to win loyalty and sincerity through their positive behavior and understanding of the organization’s concerns, such as environmental safety. When moral leaders pay attention to the employee’s issues, respect their views, listen to their concerns, help to promote ecologically, and change norms and explain moral examples, workers get suggestions from them. They can better forecast and determine the aims and organizational objectives; this means that an ecological “organizational identity” is valued and designed by ethical management (Martin et al., 2011).

Managers are highly significant promoters of ecological behavior and change in organizations. The degree of management impacts the possibility and passion of ecological change in firms, and green change will be executed in the working ability of individuals, which needs to be acknowledged by the workers (Jia et al., 2018). As per the OI, the higher the worker’s recognition with the company or the enterprise, the extra positively and willingly take action to protect the firm’s interest. When experiencing ecological pressure, managers will again redesign OI and consider environmental safety as the element of the OI (Gunarathne and Lee, 2015). Workers with a higher ecological OI will play a greater role to explore GI techniques, will apply a modern set of rules, methods, and concepts productivity to enhance the environmentally friendly manufacturing process, and will promote and generate more environmentally friendly products to attain a win-win impact of the ecological innovation action and protection. Chang found out that a more effective OI impacts positively on environmental behavior.

In contrast, the managers with a lower level of moral leadership focus extra on the presentation and are less worried about the ecological elements in decision-making (Eisenbeiß and Giessner, 2012). They may prove to be unethical managers who are not concerned about sustainable R&D, sustainable manufacturing and other ideas of morality, and a better environment, not more concerned about the ethical behaviors (Ploum et al., 2018) and goals and expense of polluting the environment with unhealthy tools and lack of good management. Such a scenario creates difficulty for the employees to trust just by the lack of individuals’ green OI. Such an environment minimizes the employee’s significant impact and response on the company in ecological change, which negatively impacts the GIB. Thus, in the way the moral management impacts employees’ GIB, only the managers with a higher ethical administration sense will surely influence the worker’s ecological OI; this will surely enable the workers to adopt the data and information achieved to green change and reaction directly to ecological problems and issues and helping in developing organization-friendly behavior. Thus, the following hypotheses were proposed:

**Hypothesis 2:** EL has a positive impact on GOI.
**Hypothesis 3:** GOI has a positive and significant influence on EGIB.
**Hypothesis 4:** GOI partial mediates between EL and EGIB.

### Strategic Flexibility and Employees’ GI Behavior

Strategic pliability is considered a strength of a firm to manage and find the solutions to modifications in the external culture (Krupskyi and Kuzmytska, 2020), modify its real plan or scheme in time, and put assets into modern initiatives to respond to modifications. Sanchez describes strategic efficiency as comprising asset efficiency and harmonized flexibility (Rodwell and Teo, 2004). Asset flexibility mirrors the specific assets, mentioned as asset investment cost, very productive value of request, and transformation cost. At the same time, harmonized flexibility mirrors the harmonized ability of firm resource allocation. In identifying the assets’ limits and finalizing and developing asset networks, planned flexibility is the quality of firms to acknowledge and manage in unsure situations (Park, 2011). Recently, most of the study majority discussed strategic efficiency as the capability of firms to respond and manage to uncertain situations and conditions. Most researchers consider planned flexibility from the firm extent. This study largely observes and discusses employees’ concept of SF, mirroring the individual’s capability to invest assets and very effective use of assets in a modifying condition (Chod et al., 2010).

When there is a transformation of the environmental protection consciousness into ecological change practices, it requires the result of all types of firms’ assets. Organizations with higher asset pliability mean lower asset septicity. Wide application category and lower cost of resources change alternatives (Cole and Bruch, 2006). The extra effective or easy the communication is, the extra efficacy of the typical enterprises will be in assigning and summarizing central and exterior assets to manage the firm inertia to cope with the ecological or green modifications (Jiang et al., 2018). The wide the enterprise’s strategic pliability in executing sustainable change, the extra it is easy to adapt to outside environmental changes. As per this aspect, staff members with a highly planned extent of perception thought that firms could invest more resources more efficiently in quite an innovative environment, which is very helpful in developing very strong enterprise activities.

In contrast, employees’ perception of SF is low, and enterprises may be unable to adapt to environmental variations and actively coordinate resources. Hence, the perception level of the SF verifies, and the behavior and the attitude of the employees will be varied (Bock et al., 2012). Employees with high SF perception levels believe that leaders cannot invest in and effectively use GI resources, worry about their role, disagree with leaders, and lack trust in leaders when dealing with environmental changes (Chen and Liu, 2018). Therefore, when the leadership motivates the subordinates to convey GIs and expects employees to engage in more green production and green R&D actively, employees will be more concerned with the organization’s strategic objectives (George et al., 2021). In this case, it is difficult for the leadership to strengthen the GOI of employees and make them produce a green presentation of innovative behavior. Therefore, we proposed the following hypotheses:

**Hypothesis 5:** SF impacts positively on EGIB.
**Hypothesis 5a:** SF as a moderator strengthens the relationship between GOI and EGIB.

**FIGURE 1:**
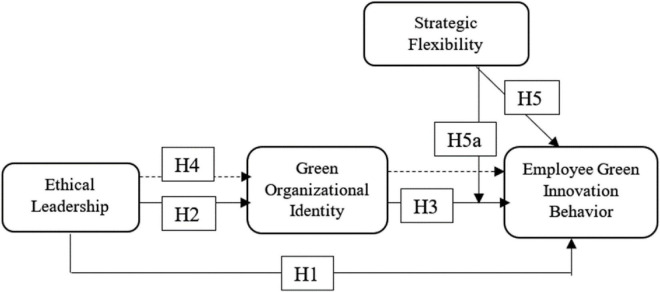
Research model.

Figure 1 shows the conceptual framework with all studied variables. The study explores the relationships and impact of EL on EGIB and GOI directly and through the mediation process. The study also explores the moderation effect of SF among GOI and EGIB.

## Materials and Methods

The researcher applied a quantitative approach to collect data from a large population. The research was conducted to analyze and test the objectives, validity, and reliability of the questionnaire and collected data. Previous research explored that quantitative research is more authentic in evaluating the relationship among variables (Creswell et al., 2003), and the applied deductive approach also tests the proposed hypotheses (Bryman, 2007). The author applied a non-probability convenience sampling technique to a Chinese small and medium enterprise (SME) employees. Chinese SMEs strive diligently to gain market share and contribute to achieving the green environmental goals. Contemporary Chinese leaders continue to rely on traditional indigenous philosophies to boost their reputations and reduce uncertainty (Li H. et al., 2021). It is crucial to investigate the EL in this study context that was not done previously. Therefore, the authors have chosen SME employees as target population of this study.

Selecting a sample size from a large population is also a crucial part of the research methodology; an inadequate sample size leads to the study’s failure or may not represent the population. The large sample size data creates a problem for a researcher in collecting and managing data, which causes extra cost timing consumption that may neglect the actual purpose of sampling (Kotrlik and Higgins, 2001). The questionnaire was initially drafted in English and was translated into Chinese language by the researchers to make it easier for the respondents to comprehend.

The cross-sectional approach was used in the study, and 450 questionnaires were distributed in December 2021 to different companies; in return, 318 responses were received and the data collection process was completed in March 2022. The last 300 responses were finalized, and 18 were discarded due to improper and missing information. The researcher completed the data collection process in different steps due to the busy schedule of the employees. The respondents were approached *via* online method of data collection from different cities in China.

The partial least-square (PLS)-structural equation modeling (SEM) technique analyzed the proposed research model using Smart-PLS v3. Smart-PLS is a powerful tool used to test mediation-moderation models and works with multivariate and normal distributions simultaneously (Hair et al., 2011). This study was conducted to explore and examine the EGIB with EL behavior. In addition, the researcher also develops confidence in the respondents about their demographic information, which will be kept secret.

### Demographics

Table 1 shows the sample statistic frequency distribution of the targeted respondents. The sample statistics include age, qualification, and job tenure. The results show that most of the respondents fall in the age group of 26–30 years old (36.67%), while 23.33% of the respondents are 31–35 years and rest in different domains. Young employees were large in number with university-level education and having 48.30%, professionals were 16.67, and 35% were graduated level. Therefore, we considered all these units for data collection, and the percentages are presented.

**TABLE 1:** Demographic information.

Particulars	Description	Frequency	Percentage
Gender	Male Female	229 71	100%
Age (in year)	21–25	35	11.67%
	26–30	110	36.67%
	31–35	70	23.33%
	36–40	40	13.33%
	47 Above	45	15%
Qualification (Educational/Professional)	Graduation level	105	35%
	University level	145	48.3%
	Professional education	50	16.67%
Job tenure	1–5 years	40	13.3%
	6–10 years	83	27.67%
	11–15 years	47	15.67%
	16–20 years	48	16%
	Above 20 years	82	27.33%

### The Measures

The objective was to measure the effectiveness and realism of the proposed model (EL, SF, GOI, and EGIB) from prior studies of 34 constructs using a 5-point Likert scale ranging from 1 strongly disagree to 5 strongly agree to quantify the results. The research applied the SEM technique through PLS—on constructed research model. SEM is applied to estimate empirical models and path correlations between latent constructs that has been widely used in different contexts (e.g., Li G. et al., 2020; Rafiq et al., 2022). Therefore, the author also applied the SEM to test the hypotheses of this study. The existing studies explored that to analyze moderation-mediation, the Smart-PLS is a powerful tool (Henseler et al., 2016). Additionally, it helps measure the validity and reliability of studies.

### Measurement Model

#### Convergent Validity and Reliability

Table 2 shows the reliability and validity of latent constructs. A test was applied to analyze and extract the factor loading of the studied variables; as per the rule of thumb, the values for factor loading should be at least 0.7 (Alghazi et al., 2021). The convergent validity with Cronbach’s alpha, rho_A, the average value extracted, composite reliability, and confirmatory factor analysis (CFA) was acceptable and above the threshold value. The values for convergent validity should be higher than the threshold values; rho_A ≥ 0.7, CR ≥ 0.8, AVE ≥ 0.50, and CA ≥ 0.80. The convergent validity for all variables is acceptable and in the range (Henseler et al., 2016).

**TABLE 2:** Convergent validity.

Variables and constructs	Loadings	CA	rho-A	CR	AVE
**Employee green innovation behavior (EGIB)**		**0.935**	**0.936**	**0.935**	**0.642**
**EGIB1**	0.729				
**EGIB2**	0.767				
**EGIB3**	0.829				
**EGIB4**	0.819				
**EGIB5**	0.773				
**EGIB6**	0.899				
**EGIB7**	0.795				
**EGIB8**	0.785				
**Strategic Flexibility (SF)**		**0.940**	**0.943**	**0.938**	**0.718**
**SF1**	0.818				
**SF2**	0.846				
**SF3**	0.916				
**SF4**	0.950				
**SF5**	0.773				
**SF6**	0.763				
**Ethical Leadership (EL)**		**0.925**	**0.927**	**0.925**	**0.500**
**EL1**	0.731				
**EL2**	0.719				
**EL3**	0.765				
**EL4**	0.742				
**EL5**	0.751				
**EL6**	0.712				
**EL7**	0.716				
**EL8**	0.700				
**EL9**	0.768				
**EL10**	0.727				
**EL11**	0.701				
**EL12**	0.744				
**EL13**	0.750				
**EL14**	0.767				
**Green Organizational Identity (GOI)**		**0.945**	**0.951**	**0.946**	**0.746**
**GOI1**	0.855				
**GOI2**	0.785				
**GOI3**	0.909				
**GOI4**	0.934				
**GOI5**	0.879				
**GOI6**	0.897				

#### Common Method Bias and Multicollinearity Test

Harman test was applied to data to perform common method bias (CMB), and variance inflation factor (VIF) factors to avoid multicollinearity. There is no issue of CMB if the merged factors are less than 50% of the variance (Harman, 1976). Therefore, the author performed a principal rotated matrix and showed that the first factor of the initial eigenvalue explains 40.24% of the total variance. Furthermore, multicollinearity has been checked using VIF, and as per the rule of thumb, the values should be less than 10, and all values were acceptable (Fornell and Larcker, 1981). In this study’s results, none of the value is above 10 (highest value was 4.906), so there is no multicollinearity issue.

#### Discriminant Validity

Fornell-Larcker criterion method was used to measure the discriminant validity and cross-loadings of latent variables (Fornell and Larcker, 1981). Table 3 shows that Fornell-Larcker criterion approach is fit to the current research, which shows there is no discriminant issue among variables.

**TABLE 3:** Fornell-Larcker criterion.

Constructs	EGIB	EL	GOI	SF
EGIB	**0.801**			
EL	0.579	**0.686**		
GOI	0.415	0.525	**0.864**	
SF	0.384	0.296	0.368	**0.847**

*Bold values are the square root of AVE.*

Table 4 shows the heterotrait-monotrait ratio (HTMT) analysis and explores the discriminant validity (Hair et al., 2011). The values for HTMT are much closer in path analysis (Henseler et al., 2016); the value for HTMT should be less than 1 among factors. Table 4 shows that all the values are in accordance with the threshold values of HTMT. Therefore, it is concluded that there is no discriminant validity issue.

**TABLE 4:** Heterotrait-Monotrait (HTMT) ratios.

Constructs	EGIB	EL	GOI	SF
EGIB				
EL	0.578			
GOI	0.410	0.525		
SF	0.382	0.293	0.370	

### Structural Model

**FIGURE 2:**
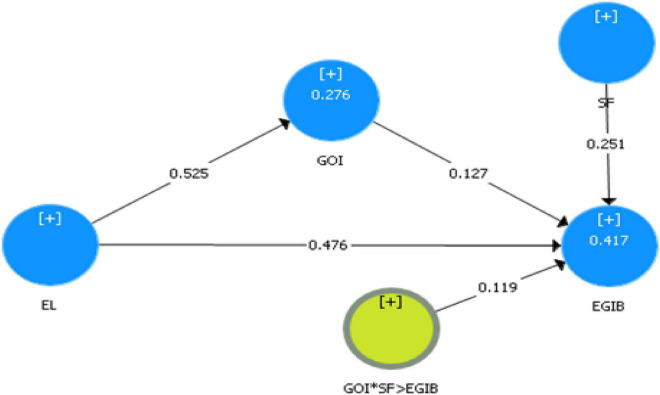
Hypotheses testing of EGIB.

Figure 2 shows factor loadings for potential constructs with acceptable values ≤ 0.70. Smart-PLS measured structural models by applying bootstrapping in 5,000 subsamples. The model was fitted with a standardized root mean square residual, and its value should be <0.08, which is a good model (McNeish et al., 2018).

Table 5 shows the direct impacts of all studied variables, H1a showed a positive and significant impact of EL on EGIB, and therefore H1 is supported (β = 0.543; *t* = 12.761; *p* < 0.000). H2 demonstrated a direct significant and positive effect of EL on GOI, and therefore H2 is supported (β = 0.525; *t* = 12.305; *p* < 0.000). H3 explained a direct and positive effect of GOI on EGIB and is supported (β = 0.127; *t* = 2.311; *p* < 0.021). H5 also showed significant, direct and positive effect of SF on EGIB, and therefore H5 is supported (β = 0.251; *t* = 5.051; *p* < 0.000).

**TABLE 5:** Path coefficients for direct relations.

Hypotheses		B	Mean	*SD*	*t*-value	*p*-value	Decision
H1	EL - > EGIB	0.543	0.545	0.043	12.761	0	Accepted
H2	EL - > GOI	0.525	0.527	0.045	12.305	0	Accepted
H3	GOI - > EGIB	0.127	0.127	0.055	2.311	0.021	Accepted
H5	SF - > EGIB	0.251	0.25	0.05	5.051	0	Accepted

Table 6 shows the indirect effects of EL through GOI as a mediator with EGIB. Furthermore, it also shows the moderating effect of SF between GOI with EGIB. The results for H4 confirm that the GOI mediates the relationship between EL and EGIB; therefore, H4 (β = 0.067; *t* = 2.187; *p* < 0.000). Moreover, this study considers the moderating effect of SF on the relationship between the GOI with EGIB. H5a shows that SF positively and significantly moderates the relationship between GOI and EGIB (β = 0.119; *t* = 4.468; *p* < 0.029).

**TABLE 6:** Indirect impacts of all studied variables.

Hypotheses		β	Mean	*SD*	*t*-value	*p*-value	Decision
H4	EL -> GOI - > EGIB	0.067	0.067	0.03	2.187	0	Accepted
H5a	GOI*SF- > EGIB	0.119	0.122	0.027	4.468	0.029	Accepted

**FIGURE 3:**
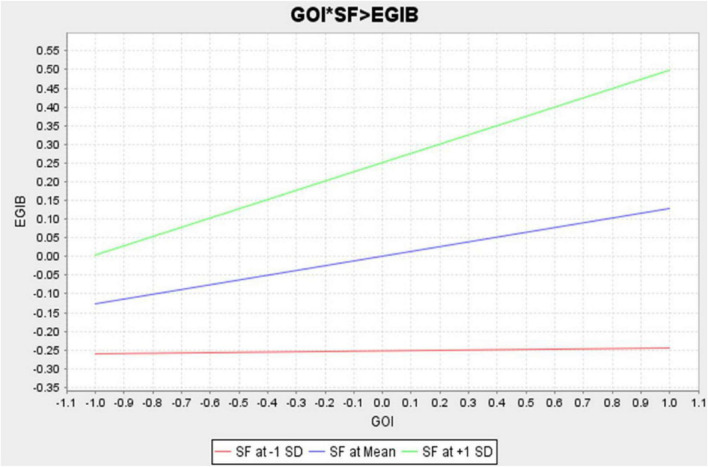
Moderating effect of SF between GOI and EGIB.

Figure 3 shows the moderating effect of SF between GOI with EGIB. Furthermore, to assess the moderating effects, other rules were applied.

Figure 3 shows that SF is an important factor that positively impacts the relationship between GOI and EGIB, proving the proposed H5a, which stated that SF has a moderating effect on the relationship between GOI and EGIB.

## Discussion

The study results in accepting the relationship of EL with EGIB and also linked through the GOI. This study also explores the moderating effect of SF between green organizational behavior and EGIB. The EL stems have the concept with the dimensions of reflective capacity and leadership. The study results provide quantitative confirmati on for the EL dimensions. Prior studies also validated the proposed model with existing models (Langlois and Lapointe, 2010).

This study contributes to and explores the role of leadership behavior on green innovative behavior, subject to the performance of employees in a firm environmental sustainability regulation. The findings show a positive and significant relationship between EL and EGIB. The results are consistent with the study that explored in different researches that the GOI mediates the relationship between EL and EGIB (Chang and Hung, 2021). Another study by Song and Yu also examined that the GOI mediates both EL and GI behavior (Song and Yu, 2018). This study identifies how leadership and EGIB legitimate each other and EL affects the GOI.

The SF plays a vital role and positively affects EGIB and moderates between green organizational identities with EGIB. However, according to the author’s expectation, it has negatively affected between EGIB and GOI. The organization with high SF tends to retain the GI behavior (Jiang et al., 2021). As the flexibility increases in the employees, different changes occur, which leads to negative and takes time to adjust resources (Boso et al., 2013).

## Conclusion and Implications

We developed a research framework involving EL and GOI to enhance GI behavior. The results showed that GOI had a direct positive effect on GI behavior. In addition, EL indirectly and positively affected EGIB through the mediating variable GOI. Therefore, the empirical results not only verify that EL is the driving factor of GOI, but also prove that GOI plays a partial mediating role between EL and EGIB. As a result, we believe that organizations should strengthen their EL, GOI, and strategic identity in order to increase their EGIB.

Furthermore, the data show that EL, GOI, SF, and EGIB of SMEs are much lower than those of large firms in China’s manufacturing industry. As a result, it is critical for SMEs in the Chinese manufacturing industry to improve their GOI, EL, and SF in order to boost their EGIB. Most of China’s SMEs have fewer resources, such that it is difficult for them to meet the SF and take advantage of green opportunities (Chen, 2011), to pay more attention to the improvement of their GOI, EL, and order to raise their EGIB.

This study contributes in several aspects. First, from the standpoint of organizational identity, the beneficial influence of green organizational identification on EGIB is investigated. To remedy the research void, we suggest a strategic GOI framework. According to research, GOI is a significant predictor of EGIB, and SF acts as a partial mediator between GOI and GI behavior. EL has an indirect favorable influence on EGIB through SF and has a positive impact on EGIB. Second, in EL, GOI, SF, and EGIB, Chinese manufacturing has a significant scale advantage. Third, utilizing the nested model and research model, the SEM findings of the research model and the nested model were compared. The use of SEM testing in this study is both suitable and adequate. Fourth, we gathered questionnaires and public data, used mixed research methodologies, and satisfied triangulation methodologically to avoid social desirability bias and the restrictions of self-reported data. This study method’s applicability is appropriate and solid.

## Data Availability Statement

The raw data supporting the conclusions of this article will be made available by the authors, without undue reservation.

## Ethics Statement

Ethical review and approval was not required for the study on human participants in accordance with the local legislation and institutional requirements. Written informed consent for participation was not required for this study in accordance with the national legislation and the institutional requirements.

## Author Contributions

LY: conceptualization, writing—original draft preparation, methodology, formal analysis, and data curation. HL: supervision, fund acquisition, project administration, writing—review and editing, and validation. Both authors contributed to the article and approved the submitted version.

## Conflict of Interest

The authors declare that the research was conducted in the absence of any commercial or financial relationships that could be construed as a potential conflict of interest.

## Publisher’s Note

All claims expressed in this article are solely those of the authors and do not necessarily represent those of their affiliated organizations, or those of the publisher, the editors and the reviewers. Any product that may be evaluated in this article, or claim that may be made by its manufacturer, is not guaranteed or endorsed by the publisher.
